# Attitudes towards the timing of first birth and gender-based violence in rural Niger: Are adolescent wives with attitudes different from their husbands and peers at a higher risk of intimate partner violence and reproductive coercion?

**DOI:** 10.1371/journal.pone.0333677

**Published:** 2025-09-30

**Authors:** Shweta Tomar, Holly Baker, Hachimou Amani, Kevin Iredell, Kadidiatou Boubacar Moussa, Abdoul-Moumouni Nouhou, Jennifer Gayles, Elizabeth Reed, Susan Kiene, Rebecka Lundgren, Jay Silverman

**Affiliations:** 1 Herbert Wertheim School of Public Health and Human Longevity Science, University of California San Diego, La Jolla, California, United States of America; 2 GRADE Africa, Niamey, Niger; 3 Save The Children, Niamey, Niger; 4 Save The Children, Washington, District of Columbia, United States of America; 5 San Diego State University, San Diego, California, United States of America; 6 Division of Global Public Health, University of California, San Diego School of Medicine, La Jolla, California, United States of America; IFPRI: International Food Policy Research Institute, UNITED STATES OF AMERICA

## Abstract

**Background:**

Intimate partner violence (IPV) and reproductive coercion (RC) significantly impact women’s and girls’ health. Among other determinants, wives’ discordance from their husbands’ fertility-related attitudes may increase husbands’ use of IPV and RC. This study aims to assess if such discordance in attitudes towards the timing of the first pregnancy is associated with IPV and RC among adolescent wives (AWs) in rural Niger.

**Methods:**

Data from 918 AWs, their husbands, and peers from a baseline assessment for a community-based family planning program in Niger were analyzed. Multilevel logistic regression models tested the association between AWs’ attitudinal discordance and their experience of IPV and RC.

**Results:**

AWs with discordant attitudes who preferred a shorter wait for childbirth than what their husbands preferred had 0.35 times lower odds of experiencing physical IPV, while those who preferred a longer wait had higher odds of experiencing sexual IPV (AOR = 2.56) and RC (AOR = 2.56). Similarly, relative to the collective attitudes of husbands in the village, the AWs with discordant attitudes supporting no delay in first childbirth had 0.23 times lower odds of experiencing physical IPV, while those who supported delayed childbirth had 6.11 times greater odds of experiencing sexual IPV.

**Conclusion:**

Violence prevention interventions need to address social norms that tie women’s values to reproductive choices. Empowering women, engaging men, and involving the community in supporting women’s autonomy in fertility decisions are crucial. Additionally, integrating IPV and RC mitigation into family planning programs is essential, especially in places like Niger, where fertility decisions are tied to cultural norms.

## Introduction

Women and girls across the world face multiple forms of gender-based violence perpetrated by their male partners, including intimate partner violence (IPV) and reproductive coercion (RC- a form of gender-based violence that includes coercing women to become pregnant or terminate a pregnancy against their will [1,2]). Global estimates on IPV show that approximately one-third of 15–49 years old ever married/partnered women face physical or sexual IPV at least once in their lifetime [3]. Research around RC in low- and middle- income countries has reported lifetime experience of RC among 10.2% of the study participants in Dosso region of Niger [2], 18.5% in rural Cote d’Ivoire [4], and 12% in rural Uttar Pradesh, India [1,5]. Another multi-country study reported past 12 months experience of RC among 20.3% in Kongo Central (DRC),16.9% in Uganda, 11.9% in Kinshasa (DRC), 7.1% in Burkina Faso, 7.0% in Kenya, 6.2% in Côte d’Ivoire, 5.7% in Kano (Nigeria), 5.0% in Lagos (Nigeria), 3.9% in Rajasthan (India) and 3.1% in Niger. Both IPV and RC are significant public health issues with noted negative impacts on women’s and girls’ health outcomes, including mental, physical, sexual, and reproductive health [1,2,5,6].

Research over the years has identified multiple determinants of IPV experience and perpetration, including poverty, child marriage, education levels, patriarchal norms, and male partners’ alcohol consumption [7–12]. The Male Backlash theory states that a man may resort to using violence against his wife if she attains more economic agency than him [13]. Based on this theory, women’s transgression from prevailing social norms, especially if the transgression challenges their husbands’ social dominance, may increase the use of violence by husbands to control their wives and maintain power within families [14–16]. Several studies across different contexts have found that women’s economic status relative to their husbands may lead to a higher risk of IPV [14,17]. For example, a study from Rwanda found that women who are employed but their husbands are not have higher odds of experiencing sexual violence [18]. Another study from Cameroon found that women’s participation in microfinance programs increased their participation in household decision-making but also increased their IPV experience, supporting the male backlash argument [19]. The relatively higher economic status of wives may provide contexts for wives to deviate from traditional gender roles, threaten men’s dominance and increase the risk of violence [13,14].

While most of the research on IPV as backlash has focused on women’s economic agency, the concept of male backlash can be applied to other spheres where women exhibit discordant attitudes and behaviors from established social norms and expectations. When applied to reproductive choices, IPV can be triggered by women’s attitudes and behaviors that challenge their husbands’ or communities’ expectations around reproductive behavior, such as pregnancy timing, family size, and contraceptive use. For example, in societies where early childbearing and high fertility are considered prestigious, women who support delayed childbearing and desire lower fertility can be seen as women with attitudinal discordance around childbearing. Building on the Male Backlash theory [13], this discordance in women’s attitudes may be perceived as a threat to their husbands’ social status and family’s reputation, leading to potential acts of violence aimed at asserting control over their wives’ decisions. For example, a multi-country study using the Demographic and Health Survey data from 32 low- and middle-income countries found that women who had a lower number of desired children than their community average, were more likely to face sexual violence perpetrated by their partners [20].

In addition to IPV, husbands may also use RC to influence their wives’ attitudes and behaviors. In instances where a wife’s fertility-related attitudes, such as the timing of the first pregnancy, deviate from her husband’s or from societal norms, men might resort to RC to assert direct control over their wives’ reproductive choices. While past research has recognized the use of IPV and RC as a means to control women’s reproductive autonomy [1,2], there is a lack of evidence on whether women’s deviance from their husbands’ attitudes or social norms around childbearing puts them at a higher risk of IPV or RC.

### Niger context

With a rate of 170 births per 1000 girls aged 15–19 years [21], Niger is a country with the highest adolescent birth rate in the world. Many girls (12.8%) in Niger become mothers before reaching the age of 15 [22]. Niger has strong pro-natal social norms which support this early initiation of childbearing [23]. Adolescent girls’ and young women’s fertility choices are often influenced by their husbands’ perceived norms [24] and social norms that link girls’ worth to their ability to bear children [25,26]. Adolescent girls who choose to delay their first birth can face social sanctions, such as being labeled infertile or disobedient [23]. Within this context of pro-natal social norms, women and girls who have attitudinal discordance around the timing of first birth may face backlash from their husbands, which can include IPV and RC. However, as stated above, there is currently a gap in evidence as no study has assessed whether women with attitudinal discordance are at a higher risk of IPV and RC. The current study aims to fill this gap by assessing whether women with discordance in attitudes towards the timing of first pregnancy are at higher risk of IPV and RC in rural Niger.

In addition to individual and dyadic (wife-husband) attitudes, “collective attitudes” refer to the prevailing norms and shared beliefs about fertility timing within a given community or village. These collective attitudes are important because they represent the broader social norms that shape individual behaviors and expectations. In many settings, including rural Niger, community norms exert strong influence over both men’s and women’s attitudes and behaviors regarding fertility. Women whose attitudes diverge from these collective norms may face disapproval from their husbands, social sanctions and increased risk of violence. Theoretically, collective attitudes may function as an independent predictor of risk, above and beyond the husband’s own attitudes, or they may shape or reinforce the husband’s attitudes, making it difficult to disentangle their effects. By examining both individual (husband-wife) and collective (village-level) discordance, our study seeks to better understand the unique influence of these social norms on adolescent wives’ risk of IPV and RC.

The specific research objectives include assessing the association of 1) adolescent wives’ (AWs’) attitudes towards the timing of first pregnancy, 2) discordance in AWs’ attitudes from their husbands’ attitudes, and 3) discordance in AWs’ attitudes from the collective attitudes among other AWs and their husbands in the village, with AWs’ IPV and RC experience. The study findings can inform the design of interventions targeted at reducing gender-based violence and can provide important considerations to reduce the risk of IPV and RC while designing family planning interventions.

## Methods

### Data

The data utilized in this study were obtained as part of the baseline study for Tipping Point (TP) evaluation, which aimed to assess the impact of a community-based family planning (FP) promotion program in the Maradi region of Niger. The TP intervention is a social network modification of a previously tested FP promotion program [27], focusing on low parity (0–1 child) married adolescent girls (15–19 years old). The study comprised of a three-arm cluster randomized control trial, including a standard (Kulawa) arm, a social network-based modification of the standard approach (TP arm), and a control arm.

The baseline data collection covered 56 villages in the Maradi region in Niger. Villages were selected and randomized through a multi-stage process, first identifying eligible villages based on the following critera:1) rural (i.e., not designated as a market town or city), 2) Hausa- or Zarma-speaking, 3) total population size of the village consistent with a population of 20–80 married, low parity (0 or 1 child) adolescent girls (age 15–19), and 4) located within 5 km of a center de santé intégré (CSI) or case de santé (CS) health facility. Out of 200 eligible villages, 51 were selected and randomly assigned to one of the three arms.

Within each village, a door-to-door house listing exercise was conducted to identify eligible AWs aged 15–19 with 0–1 child and with husbands available in the village. Interviews were conducted across the three arms with AWs and their mothers-in-law. Husbands of AWs and one alter (a close social contact) of each mother-in-law were interviewed only in the standard Kulawa and TP arms. Nine villages with fewer than 10 eligible AWs were replaced, and three additional villages were included to achieve the required sample size for the evaluation (N = 1530 wives). In total, 1538 wives (response rate = 96.5%), 1297 mothers-in-law (response rate = 89.1%), 996 husbands (response rate = 96.4%), and 822 alters (response rate = 87.6%), were interviewed between May 22^nd^ and September 15^th^, 2022. Verbal informed consent was obtained from each participant before survey administration. Participants were notified of the purpose of the study, what was entailed in their participation, risks, and benefits of participating in the study, and that participation is voluntary, consent can be withdrawn at any time during the study, and that there are no consequences for withdrawing from the study. The consent script was written in French, recorded in Hausa and Zarma, and administered verbally in the Hausa or Zarma language by trained interviews. Since almost all the village residents were non-literate, a trusted literate adult witness from the community was not available. Furthermore, a witness could compromise the confidentiality and safety of the participants and increase the risk of stigmatization. Thus, the literate adult interviewer served as a witness to the consent process. This interviewer presented the consent script orally and asked the prospective participant if they consent to study participation. Upon receiving verbal consent, the participant provided their mark of consent (a fingerprint stamp), after which the interviewer signed the consent form. This consent process was recommended by the Niger Ministry of Health Ethics Committee. Although females aged 15–17 years (minors) were included in this study, parental consent for their participation was not obtained, as all these females were married and therefore recognized as emancipated and able of providing consent for themselves in the Nigerien context. Ethics review boards of the University of California San Diego (protocol number 201921) and the Niger Ministry of Health approved all study procedures.

Since husband interviews were not conducted in control villages, we used data from only the Kulawa and TP villages for this study. In case a household had more than one eligible AW, all the eligible AWs were interviewed but only the youngest AW in the household was administered the IPV section of the survey. Out of the 1072 AWs interviewed in the Kulawa and TP villages, husbands of 62 AWs did not provide interview data. We created husband-wife dyads using data from complete interviews of both participants (N = 1010). Since 14 husbands had more than one AWs covered in the study and only the youngest AW was administered the IPV module, we dropped additional 14 dyads (with older AW) due to missing IPV data. Finally, 80 observations with missing values for the predictor or covariates were dropped resulting in the final sample of 916 AW-husband dyads. To address bias due to missing data, we did a separate analysis to assess the difference in sociodemographic variables for AWs who were dropped vs. those who were retained in the final sample. We reviewed and confirmed that all significantly different sociodemographic variables were included as covariates in all our statistical models.

### Measures

The primary study outcomes were lifetime experience of physical IPV, sexual IPV, and RC. Lifetime physical IPV was assessed using six questions, including whether the woman’s current husband had ever 1) pushed her, shook her, or thrown something at her, 2) slapped her, 3) twisted her arm or pulled her hair, 4) hit her with his fist or something that could hurt her, 5) kicked her, dragged her or beaten her, or 6) tried to choke her or burn her. Lifetime physical IPV was dichotomized as yes or no based on ‘yes’ response to any of these six items. Similarly, a woman’s experience of sexual IPV was indicated by a positive response to whether the woman’s current husband had ever done any of the following: 1) physically forced her to have sexual intercourse with him when she did not want to or 2) physically forced her to perform any sexual acts she did not want to. Both physical IPV and sexual IPV measures were drawn from the WHO Multi-country Study on Women’s Health and Domestic Violence [28].

Lifetime RC was captured using seven items validated in African context [29] and adapted from the previously published RC scale [2,30]. An AW was considered to have faced RC ever in her lifetime if her current husband had ever 1) tried to force or pressure her to become pregnant, 2) stopped her from going to a CS or CSI to obtain family planning, 3) told her that she could not use family planning because she did not have enough sons, 4) insulted her, yelled at her, or made her feel badly for using or wanting to use family planning, 5) hidden, destroyed, taken away, or demanded removal of her family planning method, 6) said that he would take another wife to have a baby with if she did not get pregnant, or 7) said he would leave her if she did not get pregnant.

Wives’ and husbands’ attitudes towards the timing of the first childbirth were captured using a single item, i.e., “In your opinion, how soon after marriage should a girl have her first child?”. The responses included “within 1st year of wedding”, “1 year after the wedding” and “at least 2 years after the wedding”. These cut-offs are consistent with results from a multi-country study in Sub-Saharan Africa (SSA) which found that more than half (56.1%) of women married before the age of 18 had a child in the first year of marriage [31]. To capture the wife’s attitudes relative to the husband’s attitudes, we created a categorical variable which was coded as “Concordant attitudes”, “AW prefers longer wait time than husband” and “AW prefers shorter wait time than husband”.

We also created two variables capturing village-level collective attitudes for wives and husbands. Wives’ collective attitude supportive of delayed childbearing was calculated as the non-self proportion of AWs in a village who supported delayed first birth, i.e., they think that a girl should have her first baby at least 1 year after the wedding. Similarly, husbands’ collective attitude supportive of delayed childbearing was calculated as the non-self proportion of husbands in the village who believe that a girl should have her first baby at least 1 year after the wedding. The non-self proportions were calculated as proportions in the village excluding the participant. Our selection of a one-year gap between marriage and the birth of the first child as the criterion for assessing village-level collective attitudes is consistent with previous research which has shown that many women in this region face significant pressure to conceive immediately after their wedding [32,33] and many child brides in SSA have a child within the first year of the wedding [31]. To capture AWs’ attitudes relative to the collective attitudes of other wives and husbands in the village, we created two categorical variables, each indicating “Concordant attitudes”, “AW with discordant attitudes favoring no delay,” and “AW with discordant attitudes favoring delay”. The categorization was based on AW’s own attitude in relation to the dominant attitude in each village (Table 1). Discordant attitudes were defined in two ways: (a) AWs who did not support delayed childbirth in villages where delaying childbirth was the dominant attitude (i.e., more than 60% of the reference group supported delay), and (b) AWs who supported delayed childbirth in villages where this was not the dominant attitude (i.e., less than 40% of the reference group supported delay). The remaining AWs, whose attitudes aligned with the prevailing community norm or did not meet criteria for discordance, were classified as having concordant attitudes.

**Table 1:** Definition of variables capturing AWs’ attitudes relative to other wives and husbands in the village.

	AW’s attitudes relative to collective attitudes of all AWs in the village	AW’s attitudes relative to collective attitudes of all husbands in the village
AW with discordant attitudes favoring no delay	AW does not support delayed childbirth and is in a village where delaying childbirth is the dominant attitude among all other AWs (i.e., more than 60% of all other AWs support delayed childbirth)	AW does not support delayed childbirth and is in a village where delaying childbirth is the dominant attitude among husbands of all other AWs (i.e., more than 60% of husbands of all other AWs support delayed childbirth)
AW with discordant attitudes favoring delay	AW supports delayed childbirth and is in a village where delaying childbirth is not the dominant attitude among all other AWs (i.e., less than 40% of all other AWs support delayed childbirth)	AW supports delayed childbirth and is in a village where delaying childbirth is not the dominant attitude among husbands of all other AWs (i.e., less than 40% of husbands of all other AWs support delayed childbirth)
Concordant attitudes	AWs who do not belong to the above categories	AWs who do not belong to the above categories

We used 40%/60% cut-offs for dominant attitudes to create clear and meaningful distinctions between villages. This approach was selected to avoid the limitations of a strict median split, which can misclassify villages whose collective attitudes are close to the midpoint and may not reflect meaningful differences. By introducing a 10% margin above and below the median, we aimed to better distinguish between villages with clearly dominant attitudes and those with more moderate or mixed views. This strategy enhances the interpretability and validity of our analyses by ensuring that the groups compared are substantively distinct.

Study covariates were selected based on their theorized association with AW’s fertility related attitudes and IPV/RC experience. These included AWs’ age, AW’s age at marriage, age difference between husband and AW, AWs’ education, husbands’ education, type of marriage, ethnicity, AWs’ parity, AWs’ employment, husbands’ employment, husbands’ migration, household asset score, and household food insecurity. Education level was categorized as 1) no school, 2) only Quranic, 3) only Modern, and 4) both Quranic and Modern. The type of marriage was categorized as monogamous vs. polygamous depending on the number of wives the husband reported. Parity was captured using the number of live births that the AW had in her life. Employment status was categorized as “worked for pay” if the participant worked for pay (in cash or in-kind) anytime over the past 12 months. Husbands who spent more than three months of the previous year outside their village were categorized as being migrants. Household asset score was measured as a sum of six items owned by the household, including (a watch, mobile phone, bicycle, motorbike or motor scooter, car or truck, and animal-drawn cart). Household food insecurity was captured as a yes/no response to the question – “In the past 30 days, did you or any members of your family go without eating the whole day because there was not enough food?”

### Analysis

We used descriptive statistics, including means and percentages, to describe the participant demographics and distribution of exposure variables for the whole sample and by categories of each outcome variable. We used T-test, Chi-square, and Fisher’s exact test (for cell value<5) to assess the differences across groups. Given the hierarchical nature of the data, we used multilevel logistic regression models to assess the relationship between each exposure variable (individual and village level attitudes and AW attitudes relative to -husband, other wives, and other husbands) and AWs’ IPV and RC experience, adjusting for all covariates and clustering on villages. The collective wives’/husbands’ attitudes variables were scaled by multiplying percentages by 10 to improve interpretability. Associations with p-value<0.05 were considered statistically significant. We utilized the Akaike Information Criterion (AIC) to assess the relative performance of our multilevel models compared to a null model for each outcome. The AIC is a measure of model fit that balances goodness of fit with the model complexity [34]. Lower AIC values indicated better models. We also tested each model’s Variance Inflation Factor (VIF) for multicollinearity. No multicollinearity was found at the VIF cutoff of four [35].

## Results

The demographic distribution of our study sample is shown in Table 2. On average, the AWs were 17.49 years old (SD = 1.34), 10.22 (SD = 7.13) years younger than their husbands and got married at age 15.40 years (SD = 1.22). The mean parity among the sampled AWs was 0.59 (SD = 0.72) children. Most (88.76%) of the AWs reported being in a monogamous relationship. Over half of the AWs and a majority of husbands (87.66%) attended any school in their life. Just one-third (32.53%) of the AWs but almost all the husbands (91.27%) worked for pay (in cash or in-kind) in the last 12 months. Almost one-third (31.88%) of the husbands migrated for a period of at least three months during the last 12 months. Most AWs and their husbands (86.46%) belonged to the Hausa ethnic group. Almost one-fifth (18.67%) of the AWs reported household food insecurity, meaning that in the last 30 days, someone in their family went without eating the whole day because there was not enough food.

**Table 2:** Sample characteristics of adolescent wives and their husbands in Maradi region of Niger (N = 916).

Continuous variables	Mean (SD)
AW’s age	17.49 (1.34)
Age difference between husband and AW	10.22 (7.13)
AW’s age at marriage	15.40 (1.22)
Parity	0.59 (0.72)
Asset score	1.76 (1.07)
**Categorical variables**	**% (n)**
Marriage type	
Monogamous	88.76 (813)
Polygamous	11.24 (103)
AW’s school	
None	41.38 (379)
Only Quranic	18.89 (173)
Only modern	17.79 (163)
Both	21.94 (201)
Husband’s school	
None	12.34 (113)
Only Quranic	33.52 (307)
Only modern	12.66 (116)
Both	41.48 (380)
AW’s work status	
No	67.47 (618)
Yes	32.53 (298)
Husband’s work status	
No	8.73 (80)
Yes	91.27 (836)
Husband’s migration status	
No	68.12 (624)
Yes	31.88 (292)
Ethnicity	
Hausa	86.46 (792)
Non-Hausa	13.54 (124)
Household food insecurity	
No	81.33 (745)
Yes	18.67 (171)

For each form of gender-based violence we measured (Table 3), approximately 5% of AW were victims, including physical IPV (5.3%), sexual IPV (4.0%), and RC (4.2%). More husbands than AWs supported delaying the first pregnancy, with 58.95% of husbands vs. 52.18% of AWs believing that a girl should have her first child after one year of the wedding and 16.59% of husbands and 9.28% of AWs believing that a girl should have her first child at least two years after the wedding. The measure for AWs’ attitudes relative to husbands’ attitudes shows that almost half (44.32%) of the AWs and their husbands had concordant attitudes. However, over one-third (36.46%) of the AWs preferred a shorter delay time from a girl’s wedding to her first childbirth than what their husbands preferred. Less than one-fifth (19.21%) of the AWs preferred a longer delay than their husbands.

The village-level collective attitudes among AWs and the husbands show that, on average, less than two-thirds (mean percentages = 61.51, SD = 18.84) of the AWs and three-fourths (mean percentages = 75.61, SD = 29.53) of the husbands in a village believed that a girl should wait for at least one year after the wedding to have her first child.

The data on AWs’ attitudes relative to the collective attitudes of other AWs in their village show that more than three-fourths (79.37%) of the AWs had concordant attitudes with other AWs in the village. Less than one-fifth (15.28%) of the AWs had discordant attitudes favoring no delay. Only 5.35% of the AWs had discordant attitudes favoring delayed childbirth, meaning that 1) they supported delayed childbirth and 2) they lived in a village where the majority (more than 60%) of the other AWs do not support delayed childbirth (at least one year after the wedding). Relative to collective attitudes among other husbands in the village, almost two-thirds (63.97%) of the AWs had concordant attitudes, 26.75% had discordant attitudes favoring no delay, and 9.28% had discordant attitudes favoring delayed childbirth. All our exposures were significantly associated with one or more of the assessed outcomes at the p < 0.05 level, except for AWs collective attitude (S1 Table).

**Table 3:** Outcome and Exposure, among adolescent wives, their husbands and collective village level in the Maradi region of Niger, 2022 (N = 916).

Outcomes	% (n)
Physical IPV	5.3 (49)
Sexual IPV	4.0 (37)
Reproductive coercion	4.2 (38)
**Exposures**	**% (n)**
AW’s attitudes towards the timing of first birth	
Within 1st year of wedding	38.54 (353)
One year after the wedding	52.18 (478)
At least 2 years after the wedding	9.28 (85)
Husband’s attitudes towards the timing of first birth	
Within 1st year of wedding	24.45 (224)
One year after the wedding	58.95 (540)
At least 2 years after the wedding	16.59 (152)
AW’s attitude relative to husband’s attitude	
Concordant attitudes	44.32 (406)
AW prefers shorter delay than husband	36.46 (334)
AW prefers longer delay than husband	19.21 (176)
	Mean (sd)
AWs’ collective attitudes supporting delayed childbearing (Scaled) ^a^	61.51 (18.84)
Husbands’ collective attitudes supporting delayed childbearing (Scaled) ^a^	75.61 (29.53)
	% (n)
AW’s attitude relative to the collective attitudes of all wives in the village	
Concordant attitudes	79.37 (727)
AW with discordant attitudes favoring no delay	15.28 (140)
AW with discordant attitudes favoring delay	5.35 (49)
AW’s attitude relative to the collective attitudes of all husbands in the village	
Concordant attitudes	63.97 (586)
AW with discordant attitudes favoring no delay	26.75 (245)
AW with discordant attitudes favoring delay	9.28 (85)

^a^The proportion of wives or husbands in the village who think the first pregnancy should be at least 1 year after the wedding.

The results from Table 4 show that AWs’ attitude towards the timing of first birth was associated with their experience of physical and sexual IPV but not with RC. Compared to the AWs who believed that a girl should have her first child within the first year of the wedding, those who thought that a girl should have her first child at least 1 year after the wedding had 2.84 (95% CI = 1.25,6.46) times greater odds of experiencing physical IPV and 2.62 (95% CI = 1.03,6.71) times greater odds of experiencing sexual IPV. This association was stronger for longer delay. The AWs who believed that a girl should have her first child at least 2 years after the wedding had 4.00 (95% CI = 1.28,12.48) times greater odds of experiencing physical IPV and 4.21 (95% CI = 1.14,15.59) times greater odds of experiencing sexual IPV.

**Table 4:** Logistic regression results for associations between forms of violence experienced and relevant outcomes among adolescent wives in the Maradi region of Niger, 2022 (N = 916).

	Physical IPV		Sexual IPV		Reproductive coercion	
	AOR (95% CI)	AIC	AOR (95% CI)	AIC	AOR (95% CI)	AIC
**Null model**		383.4		318.3		321.8
AW’s attitudes towards the timing of first birth						
Within 1st year of wedding	Ref	378.9	Ref	316.1	Ref	322.8
One year after the wedding	2.84* **(1.25-6.46)**		2.62* **(1.03-6.71)**		2.08^ (0.89-4.82)	
At least 2 years after the wedding	4.00* **(1.28-12.48)**		4.21* **(1.14-15.59)**		1.62 (0.42-6.34)	
Husband’s attitudes towards the timing of first birth						
Within 1st year of wedding	Ref	383.0	Ref	317.3	Ref	321.3
One year after the wedding	0.81 (0.34-1.94)		0.39* **(0.17-0.93)**		0.38* **(0.15-0.96)**	
At least 2 years after the wedding	0.29^ (0.08-1.09)		0.29^ (0.08-1.01)		0.76 (0.26-0.26)	
AW’s attitude relative to husband’s attitude						
Concordant attitudes	Ref	378.2	Ref	312.7	Ref	320.7
AW prefers shorter delay than husband	0.34* **(0.15-0.81)**		0.52 (0.19-1.46)		1.12 (0.47-2.69)	
AW prefers longer delay than husband	1.35 (0.63-2.88)		2.56* **(1.14-5.77)**		2.53* **(1.02-6.27)**	
AWs’ collective attitudes supporting delayed childbearing (Scaled) ^a^	1.23^ (0.96-1.58)	382.8	0.95 (0.74-1.24)	320.2	1.05 (0.79-1.38)	323.7
Husbands’ collective attitudes supporting delayed childbearing (Scaled) ^a^	0.85* **(0.74-0.97)**	380.2	0.78*** **(0.69-0.88)**	304.9	0.83* **(0.70-0.97)**	318.4
AW’s attitude relative to the collective attitudes of all wives in the village						
Concordant attitudes	Ref	381.3	Ref	314.7	Ref	322.3
AW with discordant attitudes favoring no delay	0.25* **(0.07-0.90)**		0.31 (0.07-1.40)		0.33 (0.09-1.24)	
AW with discordant attitudes favoring delay	0.96 (0.18-5.08)		3.76* **(1.09-13.01)**		1.35 (0.31-5.79)	
AW’s attitude relative to the collective attitudes of all husbands in the village						
Concordant attitudes	Ref	376.8	Ref	304.3	Ref	323.5
AW with discordant attitudes favoring no delay	0.23** **(0.08-0.71)**		0.26^ (0.06-1.15)		0.53 (0.18-1.56)	
AW with discordant attitudes favoring delay	2.02 (0.67-6.10)		6.11*** **(2.19-17.06)**		1.77 (0.47-6.72)	

^a^The proportion of wives or husbands in the village who think the first pregnancy should be at least 1 year after the wedding.

^/*/**/*** imply statistically significant association at p < 0.10/0.05/0.01/0.001 level

Husbands’ attitudes were associated with AWs’ reports of sexual IPV and RC but not with physical IPV. Compared to the AWs whose husbands thought that a girl should have her first child within the first year of the wedding, the AWs whose husbands thought that a girl should wait for at least one year after the wedding had lower odds of experiencing sexual IPV (AOR = 0.39; 95% CI = 0.17,0.93) and RC (AOR = 0.38; 95% CI = 0.15,0.96).

Results from the model with AWs’ attitudes relative to the attitudes of their husbands show that while the discordance between AWs’ and their husbands’ attitudes was associated with all three forms of violence, the nature of this association differed for physical IPV compared to sexual IPV and RC. Compared to AWs with concordant attitudes, those who believed that the wait time for first childbirth should be shorter than what their husbands thought, had lower odds of experiencing physical IPV (AOR = 0.34; 95% CI = 0.15, 0.81). AWs’ attitudes supportive of longer delay than their husbands were not associated with physical IPV. However, compared to the AWs with concordant attitudes, those who believed that the wait time should be longer than what their husbands thought, had greater odds of experiencing sexual IPV (AOR = 2.56; 95% CI = 1.14, 5.77) and RC (AOR = 2.56; 95% CI = 1.14, 5.77). No association was observed between AWs’ attitudes supportive of shorter delay than their husbands and their experience of sexual IPV or RC.

The results from the model with collective attitudes as predictors show that AWs’ collective attitudes regarding the timing of first birth were not associated with any form of violence reported by individual AWs. However, husbands’ collective attitudes at the village level were negatively associated with all three forms of violence. With an increase of 10% in the proportion of husbands who believe that a girl should have her first child at least one year after the wedding, the odds of AWs’ reporting physical IPV, sexual IPV, and RC was lower by 0.85 (95% CI = 0.74, 0.97), 0.78 (95% CI = 0.69, 0.88), and 0.83 (95% CI = 0.70, 0.97) times, respectively.

Finally, we assessed the association of AWs’ attitudinal discordance from the collective attitudes among other AWs and other husbands in their village with the AWs’ experience of IPV and RC. The results show that compared to other AWs, those with discordant attitudes supporting delayed childbirth had greater odds of experiencing sexual violence (AOR = 3.76; 95% CI = 1.09, 13.01). This means that, compared to AWs with concordant attitudes, an AW was at a higher risk of sexual IPV if she supported delayed childbirth but lived in a village where the majority (above 60%) of other AWs did not support delayed childbirth. AWs’ discordance from husbands’ collective attitudes was associated with physical and sexual IPV but not with RC. The AWs with discordant attitudes supporting no delay had lower odds of experiencing physical IPV (AOR = 0.23; 95% CI = 0.08, 0.71), while those with discordant attitudes supporting delayed childbirth had greater odds of experiencing sexual IPV (AOR = 6.11; 95% CI = 2.19, 17.06).

The VIF value for all the models was less than 4, indicating no multicollinearity. The AIC values for null models were 383.5, 318.3, and 321.8 for physical IPV, sexual IPV, and RC, respectively. AIC values for individual models are reported in Table 4.

## Discussion

The current study provides novel findings on the association of spousal and community dynamics around fertility-related attitudes and young women’s experience of gender-based violence in rural Niger. To our knowledge, this is the first study assessing the association of AWs’ discordance from their husbands’ and collective peer attitudes towards the timing of first childbirth with AWs’ experience of IPV and RC. The study found that AWs’ discordant attitudes favoring delayed childbirth is associated with higher odds of experiencing sexual IPV and RC. However, AWs’ discordant attitudes against delayed childbirth is associated with lower odds of experiencing physical IPV.

Our results show that the proportion of AWs experiencing the three forms of partner-perpetrated gender-based violence is relatively low in Niger. Globally, 24% of adolescent girls (aged 15–19 years) are estimated to have experienced physical or sexual IPV in their lifetime [36]. Compared to these global estimates, the AWs’ reports of physical (5%) and sexual (4%) IPV are notably lower than in other contexts. However, our prevalence rates closely align with the national estimate of IPV among adolescent wives in Niger, which stands at 8.5% for the lifetime experience of physical IPV and 5.4% for sexual IPV [37]. The RC prevalence rate is also lower in our sample compared to the previous study conducted in the Dosso region of Niger [2]. However, it is essential to highlight that our study takes place in a different region of Niger, which might have a different social environment.

The distribution of measures for individual, collective, and discordant attitudes towards the timing of first childbirth provides critical insights into the spousal and community dynamics around fertility in Niger. The results indicate a substantial variance between the opinions of husbands and AWs regarding the optimal wait time after marriage for a girl to have her first child. Notably, a higher percentage of husbands overall and within individual villages (collective husbands’ attitudes) expressed support for delaying the first pregnancy compared to AWs. These differences could be partially explained by the societal emphasis on women’s fertility as a measure of their worth [25,26]. In societies with high levels of gender inequality, such as Niger, women face greater sanctions than men and are blamed for failing to bear children [38–40]. Under these circumstances, women might feel compelled to prove their fertility earlier, internalizing societal expectations and fearing social sanctions. These dynamics highlight the need to address gender-specific social expectations around fertility in the context of Niger. Despite some degree of discordance, most wives had attitudes concordant with collective attitudes among other AWs and husbands.

The association between attitudes towards delayed childbearing and increased risk of IPV suggests a backlash mechanism, aligning with theories of male control. In Niger’s context, with its high adolescent birth rates and strong pro-natal norms, our findings are particularly salient, pointing towards social sanctions extending into the domain of intimate partner violence. The negative association of AW’s attitudes supporting a shorter delay in childbirth than their husband with physical IPV reflects the protective effects of adhering to social norms on AW’s physical IPV experience. In contexts where early childbirth aligns with prevailing social norms, an AW’s preference for a shorter delay may signal conformity with gender expectations, possibly resulting in greater marital harmony. Moreover, when AWs support norm-consistent behaviors, husbands may feel less pressure to enforce traditional roles, reducing the risk of IPV. Furthermore, the positive association of AWs’ attitudes supporting longer delay in childbirth than their husbands with sexual IPV and RC, rather than physical IPV, may reflect the direct relevance of these types of violence to fertility and reproductive control. Sexual IPV and RC are more directly linked to reproductive autonomy and decisions, suggesting that when women’s fertility attitudes diverge from their husbands’, they may be more susceptible to coercion and violence aimed at controlling their reproductive choices. It is also important to note the possibility that AWs facing IPV/RC may develop attitudes supporting delayed childbirth instead of these attitudes resulting in IPV/RC as backlash.

At the village level, the association of collective husbands’ attitudes, rather than collective AWs’ attitudes, with all forms of violence indicates that at the community level, men’s ideas around fertility are more critical in determining violence perpetration against AWs. This finding underscores the importance of collective attitudes—not merely as a background context, but as an active influence on individual behavior. Collective attitudes may function both as independent predictors of violence and as drivers that shape individual husbands’ beliefs and actions through mechanisms such as social pressure, normative expectations, and the desire to conform to prevailing community standards.

Finally, the association of discordance between AW’s and husband’s attitudes with RC, but no association of discordance between AW’s and collective husbands’ attitudes with RC, indicates that RC may be a more individualized form of control. This suggests that while community norms influence overall attitudes towards fertility, enforcing these norms within a marriage, especially in the form of RC, is more closely tied to the dynamics between individual couples. It underscores the importance of understanding and addressing the interpersonal aspects of RC at the couple level within the context of broader societal norms. However, it should also be noted that these couple-level dynamics are also influenced by social norms therefore, modifying broader social norms can also have substantial downstream impacts on the interpersonal aspects of RC.

While our study provides critical insights, it is not without limitations. The cross-sectional nature of the data limits our ability to infer causality. It may not be that violence emerges as a backlash to women’s fertility intentions; instead, the coercive nature of a relationship itself may shape a woman’s fertility choices. The study’s reliance on self-reported data introduces potential biases, especially for IPV and RC, as such experiences are often underreported due to stigma or fear of retribution. The low prevalence of outcomes, specifically sexual IPV and RC resulted in relatively low power to detect smaller effects. The RC scale, while validated in other African context, the adapted version used in the current study was not validated in Niger. Our study is constrained by its scope of comparison, focusing solely on women’s attitudes in relation to their peers and husbands. This approach overlooks the potentially influential roles of other significant figures, such as parents, mothers-in-law, and community elders. Although data on mothers-in-law were collected, a detailed examination of their influence was beyond the scope and length constraints of this manuscript. To further understand these dynamics, our future research will specifically investigate the roles of mothers-in-law and other significant actors within these social networks. The methodology involving cutoff of 40% and 60% to categorize community level deviance has not been tested previously. Finally, our study’s focus on low-parity AWs in rural Niger may limit the generalizability of the findings to other contexts or populations.

This study has several notable strengths. Its use of dyad-level data—capturing linked responses from both AWs and their husbands—enables a nuanced analysis of attitudinal discordance and its associations with key outcomes. Additionally, the integration of both individual- and community-level measures allows for a comprehensive assessment of how collective attitudes and interpersonal dynamics effect gender-based violence experienced by AWs in high fertility contexts like rural Niger. The inclusion of villages with varying fertility-related norms strengthened the study by capturing a broad range of cultural perspectives.

## Conclusion

The findings of this research have important implications for designing interventions to reduce gender-based violence. Interventions need to be sensitive to the cultural context and address the underlying social norms that link a woman’s value to her reproductive choices. Such interventions should aim to not only empower women but also engage men and the broader community to foster a more supportive environment for women’s autonomy in fertility decisions. Evidence shows that interventions using entertainment media and those working with champions have been effective in shifting social norms around promoting family planning use and supporting women’s reproductive autonomy [41]. This study underscores the necessity for comprehensive family planning interventions that integrate components to mitigate the risk of IPV and RC. This approach is crucial in contexts like Niger, where fertility decisions are deeply embedded in social and cultural norms.

## Supporting information

S1 Table Outcome and Exposure- Total and by forms of violence experienced, among adolescent wives, their husbands and collective village level in the Maradi region of Niger, 2022 (N = 916). (DOCX)

S1 File Study data. (XLSX)
