# Supplementary material for: Attitudes towards the timing of first birth and gender-based violence in rural Niger: Are adolescent wives with attitudes different from their husbands and peers at a higher risk of intimate partner violence and reproductive coercion?
Source: PLoS One. 2025 Sep 30;20(9):e0333677. doi: 10.1371/journal.pone.0333677 (PMC12483244; doi:10.1371/journal.pone.0333677)
Supplement: S1 Table — (DOCX) [file pone.0333677.s001.docx]

| **Table S1: Outcome and Exposure- Total and by forms of violence experienced, among adolescent wives, their husbands and collective village level in the Maradi region of Niger, 2022 (N=916)** | | | | | | | | | | |
| --- | --- | --- | --- | --- | --- | --- | --- | --- | --- | --- |
|  | **Total** | **Physical IPV** | | | **Sexual IPV** | | | **Reproductive coercion** | | |
|  |  | **No** | **Yes** | **p-value** | **No** | **Yes** | **p-value** | **No** | **Yes** | **p-value** |
|  | N=916 | N=867 | N=49 |  | N=879 | N=37 |  | N=878 | N=38 |  |
|  | 100% | 94.7% | 5.3% |  | 95.9% | 4.0% |  | 95.8% | 4.2% |  |
|  | **% (n)** | **% (n)** | **% (n)** |  | **% (n)** | **% (n)** |  | **% (n)** | **% (n)** |  |
| AW’s attitudes towards the timing of first birth | | | | | | | | | | |
| Within 1st year of wedding | 38.54 (353) | **39.68 (344)** | **18.37 (9)** | **<0.01** | **39.36 (346)** | **18.92 (7)** | **0.02** | 39.07 (343) | 26.32 (10) | 0.27 |
| One year after the wedding | 52.18 (478) | **51.44 (446)** | **65.31 (32)** |  | **51.65 (454)** | **64.86 (24)** |  | 51.71 (454) | 63.16 (24) |  |
| At least 2 years after the wedding | 9.28 (85) | **8.88 (77)** | **16.33 (8)** |  | **8.99 (79)** | **16.22 (6)** |  | 9.23 (81) | 10.53 (4) |  |
| Husband’s attitudes towards the timing of first birth | | | | | | | | | | |
| Within 1st year of wedding | 24.45 (224) | 23.99 (208) | 32.65 (16) | 0.16 | **23.44 (206)** | **48.65 (18)** | **<0.01** | **23.58 (207)** | **44.74 (17)** | **<0.01** |
| One year after the wedding | 58.95 (540) | 58.94 (511) | 59.18 (29) |  | **59.73 (525)** | **40.54 (15)** |  | **60.02 (527)** | **34.21 (13)** |  |
| At least 2 years after the wedding | 16.59 (152) | 17.07 (148) | 8.16  (4) |  | **16.84 (148)** | **10.81 (4)** |  | **16.40 (144)** | **21.05 (8)** |  |
| AW’s attitude relative to husband’s attitude | | | | | | | | | | |
| Concordant attitudes | 44.32 (406) | **43.94 (381)** | **51.02 (25)** | **<0.01** | **44.60 (392)** | **37.84 (14)** | **<0.01** | **44.76 (393)** | **34.21 (13)** | **0.02** |
| AW prefers shorter delay than husband | 36.46 (334) | **37.60 (326)** | **16.33 (8)** |  | **37.32 (328)** | **16.22 (6)** |  | **36.79 (323)** | **28.95 (11)** |  |
| AW prefers longer delay than husband | 19.21 (176) | **18.45 (160)** | **32.65 (16)** |  | **18.09 (159)** | **45.95 (17)** |  | **18.45 (162)** | **36.84 (14)** |  |
|  | Mean (sd) | Mean (sd) | Mean (sd) |  | Mean (sd) | Mean (sd) |  | Mean (sd) | Mean (sd) |  |
| AWs’ collective attitudes supporting delayed childbearing (Scaled) ^a^ | 61.51 (18.84) | **61.08 (18.78)** | **69.16 (18.42)** | **<0.01** | 61.51 (18.82) | 61.35 (19.62) | 0.96 | 61.42 (18.85) | 63.59 (18.78) | 0.49 |
| Husbands’ collective attitudes supporting delayed childbearing (Scaled) ^a^ | 75.61 (29.53) | **76.17 (28.83)** | **65.66 (39.05)** | **0.07** | **76.76 (28.49)** | **48.40 (39.81)** | **<0.01** | **76.29 (29.09)** | **59.98 (35.25)** | **0.01** |
|  | % (n) | % (n) | % (n) |  | % (n) | % (n) |  | % (n) | % (n) |  |
| AW’s attitude relative to the collective attitudes of all wives in the village | | | | | | | | | | |
| Concordant attitudes | 79.37 (727) | 78.78 (683) | 89.80 (44) | 0.15 | **79.41 (698)** | **78.38 (29)** | **0.01** | 79.16 (695) | 84.21 (32) | 0.36 |
| AW with discordant attitudes favoring no delay | 15.28 (140) | 15.80 (137) | 6.12  (3) |  | **15.70 (138)** | **5.41 (2)** |  | 15.60 (137) | 7.89 (3) |  |
| AW with discordant attitudes favoring delay | 5.35 (49) | 5.42 (47) | 4.08  (2) |  | **4.89 (43)** | **16.22 (6)** |  | 5.24 (46) | 7.89 (3) |  |
| AW’s attitude relative to the collective attitudes of all husbands in the village | | | | | | | | | | |
| Concordant attitudes | 63.97 (586) | **63.32 (549)** | **75.51 (37)** | **<0.01** | **64.16 (564)** | **59.46 (22)** | **<0.01** | **63.55 (558)** | **73.68 (28)** | 0.11 |
| AW with discordant attitudes favoring no delay | 26.75 (245) | **27.80 (241)** | **8.16 (4)** |  | **27.65 (243)** | **5.41 (2)** |  | **27.33 (240)** | **13.16 (5)** |  |
| AW with discordant attitudes favoring delay | 9.28 (85) | **8.88 (77)** | **16.33 (8)** |  | **8.19 (72)** | **35.14 (13)** |  | **9.11 (80)** | **13.16 (5)** |  |
| ^a^ The proportion of wives or husbands in the village who think the first pregnancy should be at least 1 year after the wedding. | | | | | | | | | | |
